# Effect of exogenous gonadotropin on the transcriptome of human granulosa cells and follicular fluid hormone profiles

**DOI:** 10.1186/s12958-019-0489-4

**Published:** 2019-06-24

**Authors:** Cui-Ling Lu, Zhi-Qiang Yan, Xue-Ling Song, Yang-Ying Xu, Xiao-Ying Zheng, Rong Li, Ping Liu, Huai-Liang Feng, Jie Qiao

**Affiliations:** 1 Center for Reproductive Medicine, Department of Obstetrics and Gynecology, Peking University Third Hospital, Beijing, 100191 China; 2 National Clinical Research Center for Obstetrics and Gynecology, Beijing, 100191 China; 3 Key Laboratory of Assisted Reproduction, Ministry of Education, Peking University, Beijing, 100191 China; 4 Beijing Key Laboratory of Reproductive Endocrinology and Assisted Reproductive Technology, Beijing, 100191 China; 5 Peking-Tsinghua Center of Life Sciences, Peking University, Beijing, 100871 China; 6 Beijing Advanced Innovation Center for Genomics, Beijing, 100871 China; 7 Department of Obstetrics and Gynecology, New York Hospital Queens-affiliated Weill Medical College, Cornell University, New York, 10041NY212 USA

**Keywords:** Granulosa cells, Transcriptome, Follicular fluid, Gonadotropins, Natural cycle

## Abstract

**Background:**

Superovulation treatment had some adverse effects on maturity and development of oocytes. Can superovulation dose of gonadotropins (Gns) affect the transcriptome of granulosa cells and follicular fluid (FF) hormone levels?

**Methods:**

One leading pre-ovulatory follicle per subject was used from three natural-cycle and four Gn-stimulated patients. Granulosa cells and FF samples were collected from the same leading follicle of each patient. RNA was extracted from granulosa cells and subjected to deep sequencing and analysis. Follicle-stimulating hormone (FSH), estradiol (E2), androstenedione (AND), testosterone (T), luteinizing hormone (LH), and progesterone (P4) levels in FF were measured by immunoassays. Student’s t test was used for statistical analysis.

**Results:**

A total of 715 genes were up-regulated, and 287 genes were down-regulated, in the Gn-stimulated group relative to the control group. Gene Ontology analysis revealed that the down-regulated genes were enriched in cell cycle and meiosis pathways, primarily those associated with follicle or oocyte maturation and quality. On the other hand, the up-regulated genes were enriched in functions related to immunity and cytokine–cytokine receptor interactions. Compared to the follicles of natural cycle, the E2 and LH concentrations were significantly reduced (*P* < 0.001), the P4 concentration was significantly increased (*P* = 0.003), and the concentrations of FSH, T and AND had no difference in the follicles of Gn-stimulated cycle.

**Conclusions:**

Cell cycle– and meiosis-associated genes were down-regulated by Gns stimulation, whereas immune- and cytokine-associated genes were up-regulated. Hormone levels were also affected by Gns stimulation. Compared with natural-cycle follicles,putative markers associated with oocyte quality and follicle maturation were significantly different from those in Gn-stimulated follicles. Hormone levels in follicles were compatible with the steroidogenic patterns of granulosa cell, which reflects the follicle maturation and oocyte quality.

**Electronic supplementary material:**

The online version of this article (10.1186/s12958-019-0489-4) contains supplementary material, which is available to authorized users.

## Background

Controlled ovarian stimulation (COS) using exogenous gonadotropins (Gns) is a conventional step of the in vitro fertilization (IVF) treatment. With regard for this technique, oocyte retrieval is performed with an aim to harvesting a large number of oocytes to achieve a successful pregnancy (i.e., the more oocytes obtained per cycle, the more embryos can be selected for transfer) [1]. In recent years, the concept of acquiring as many oocytes as possible has been shifted to place emphasis on obtaining a cohort of high-quality embryos [2]. Although various COS protocols have been proposed to achieve a large number of oocytes and subsequently developed embryos, there is no evidence to support an appropriate and optimal protocol that obtains the best quality of products.

Additionally, COS may result in the recruitment of oocytes that are not reaching their optimal maturation or full competency, and thus are not the best quality of oocytes and embryos compared to those obtained via the natural cycle [3–5]. Studies performed using clinical samples have demonstrated that compared with conventional IVF cycle, treatment using in natural cycle IVF without embryo selection achieved a higher implantation rate [6, 7]. Furthermore, ovarian stimulation with a higher dose of Gns can lead to an iatrogenic complication called ovarian hyperstimulation syndrome (OHSS), which can be life-threatening. Moreover, offspring of women who experience OHSS has been reported to exhibit a higher chance of diminished intellectual ability and cardiovascular dysfunction [8, 9].

Collectively, IVF performed using natural cycle protocol may provide a superior environment for oocyte maturation. Therefore, the application of COS protocol for IVF may have a detrimental effect on the follicular milieuand hence an impact on the maturation process and developmental competence of oocytes. In this study, we aimed to investigate the endocrine milieu in the nature-cycle follicle and the COS-follicle by comparing the granulosa cells and hormonal composition in the follicle fluid samples of two conditions during the time of follicular aspiration.

## Methods

### Study design, participants and collection of granulosa cells

The study was conducted at the Reproductive Medical Center of the Third Hospital Peking University (Beijing, China). All the recruited patients were under 36 years old and had a normal BMI range from 18.8 to 26.6 kg/m^2^. These patients received either the Gn stimulation protocol (the same COS protocol) or natural cycle for their IVF treatment, as explained below. The exclusion criteria were patients with the ovulatory disorder or follicular dysplasia. Four women who received the COS protocol for IVF treatment had a regular menstrual cycle because of male factor or tubal factor. For these patients, a combination of recombinant follicle stimulating hormone (FSH) (Gonal-F, 150 IU/day, Merck Serono SA Aubonne Branch) and human menopausal gonadotrophin (Menotropins for injection FSH 75 IU: LH 75 IU, Livzon Pharmaceutical Group Inc.) in a fixed-dose was started on Day 2 of the menstrual cycle with the option to adjust dose according to response after 4 days of stimulation (Day 6 of menstrual cycle). GnRH antagonist (Cetrorelix 250 μg/day, Merck Serono, Darmstadt, Germany) was started when a leading follicle of 12 mm was achieved. Moreover, when one or two leading follicles reached an average diameter of 18 mm, the recombinant human chorionic gonadotrophin (hCG) (Ovitrelle, 250 μg; Merck Serono S.p.A, Rome, Italy) was administered followed by follicular aspiration 34–36 h later. These patients were assigned as the Gn-stimulated group (*n* = 4). In the unstimulated-cycle group (assigned as the control group, *n* = 3), three patients were arranged for IVF treatment with their natural cycles without Gn intervention and with no hCG triggering. These infertile women were due to tubal factors, including hydrosalpinx and proximal tubal obstruction. In both groups, the follicular fluid samplesobtained from the largest pre-ovulatory follicle of each patient were collected for analysis. The technique of follicular aspiration was performed with a new 17-gauge single-lumen aspiration needle (K-OPS-7035-REH-ET; Cook, Queensland, Australia) and a suction pressure of 120 mmHg, under the guidance with transvaginal ultrasonography. Immediately after the aspirates were collected, we centrifuged these aspirates at 2000Xg for 10 min, and the supernatant was transferred to a 2-ml cryotube for cryopreservation at − 80 °C until later analysis. The sediment samples were collected for granulosa cell isolation, which were further washed in phosphate-buffered saline (PBS) and centrifuged over Ficoll (GE Healthcare Corp., USA) to remove the red blood cells. The aggregates that contain granulosa cells were isolated from the follicular fluid by a pipette, flushed twice in phosphate-buffered saline (PBS), and transferred to a tube placed in ice water. Granulosa cells were washed again with PBS, and the cell deposits were flash frozen in liquid nitrogen within 30 min and stored at − 80 °C until RNA extraction.

### Preparation of cDNA from a small number of cells and PCR pre-amplification

The experimental protocol for cDNA preparation and PCR pre-amplification was as previously described [10]. Briefly, deposits of a small amount of granulosa cells were transferred into lysis buffer and reversely transcribed into the first-strand cDNA. The cDNA was then amplified by a PCR machine for 20 cycles.

### Tagmentation reaction and final PCR amplification

PCR was purified and ultimately eluted. Five nanograms of cDNA were then used for the tagmentation reaction, purified, and then used for second PCR amplification. The PCR products were purified using an AMPure XP kit (Beckman Coulter, Brea, CA, USA), and samples were loaded onto an Agilent Bioanalyzer 2100 system (Agilent Technologies, Santa Clara, CA, USA). Quantification of the library was performed using the Qubit 3.0 Fluorometer (Thermo Fisher Scientific, Singapore). Libraries were diluted to a final concentration of 2 nM, and a total of 10 pmol of each library was sequenced using an Illumina HiSeq 2000.

### Read alignments and estimation of gene expression

Clean data (clean reads) were obtained by removing artificial adapters, poly-A, and low-quality bases from raw data. Clean data were aligned to human (hg19) genome using Tophat2 (v2.1.0) with default settings and filtered for uniquely mapped reads. Gene expression values were calculated as FPKM using Cufflinks (v2.2.1) [11].

### Data analysis

Differential expression analysis of the two groups was performed using the DEGSeq R package (v1.18.0). DEGSeq analysis was used to provide statistical routines for determining differentially expressed genes (DEGs) using a model based on the negative binomial distribution. *P*-values were adjusted using the Benjamini–Hochberg method to control the false discovery rate. Genes with adjusted *P*-value < 0.05 were defined as differentially expressed.

Gene Ontology (GO) enrichment analysis was performed using the GOseq R package with correction for gene length bias. GO terms with corrected *P*-values less than 0.05 were considered to be significantly enriched. Enrichment of DEGs in the Kyoto Encyclopedia of Genes and Genomes (KEGG) was analyzed using the KOBAS software [12].

### Hormone assay procedures

All hormonal assays were performed at the endocrine laboratory of the Peking University Third Hospital Reproductive Centre using commercially available ELISA kits (IMMULITE 2000, SIEMENS, USA). The lower detection limits of the six assays used in this study were as follows: 0.1 mIU/ml for FSH, 0.1 mIU/ml for luteinizing hormone (LH), 73.4 pmol/l for estradiol (E2), 0.64 nmol/l for progesterone (P4), 0.69 nmol/l for testosterone (T), and 1.05 nmol/l for androstenedione (AND), respectively. The inter-assay coefficients of variation (CVs) for the six hormone levels were 5% (FSH), 6% (LH), 5% (E2), 6% (P), 6% (T), and 5% (AND), respectively.

### Statistical analysis

Hormonal concentration valuesare expressed as means ± SD (standard deviation). Differences in hormone concentration between the two groups were analyzed by student’s t test.

## Results

### Samples and sequencing

The characteristics of the patients of the control group (*n* = 3) and Gn-stimulated group (*n* = 4) were as the previous described in the Materials and Methods section. Transcriptomes of the individual luteinized granulosa cells from every single pre-ovulatory follicle obtained from these seven women were sequenced.

RNA transcriptomes were sequenced using the Illumina HiSeq platform. We obtained 69–90 million 150 bp reads for each sample, and 76 Gb of raw data in total were obtained for all of the samples. Clean data were ~ 10 Gb per sample (Additional file 2: Table S1). All the downstream analyses were conducted based on clean, high-quality data.

### Transcriptional profiles between different samples

We calculated the Pearson correlation coefficient of every two samples by using the gene expression in the samples. As shown in Additional file 1: Figure S1A, the expression patterns were generally homogeneous among the three controls (R^2^ > 0.9 for both comparisons). In contrast, the expression patterns were relatively heterogeneous in the Gn-stimulated group, with Gns1 and Gns3 (R^2^ = 0.917), and Gns2 and Gns4 (R^2^ = 0.841), are more similar to each other.

We then examined the total number of transcripts that were expressed in each group. The results obtained from the comparison analyses revealed that 11,923 genes were commonly expressed in both groups in which 2437 genes were expressed uniquely in the Gn-stimulated group and 920 genes were uniquely expressed in the control group (Additional file 1: Figure S1B).

### DEGs and function enrichment analysis

**Fig. 1:**
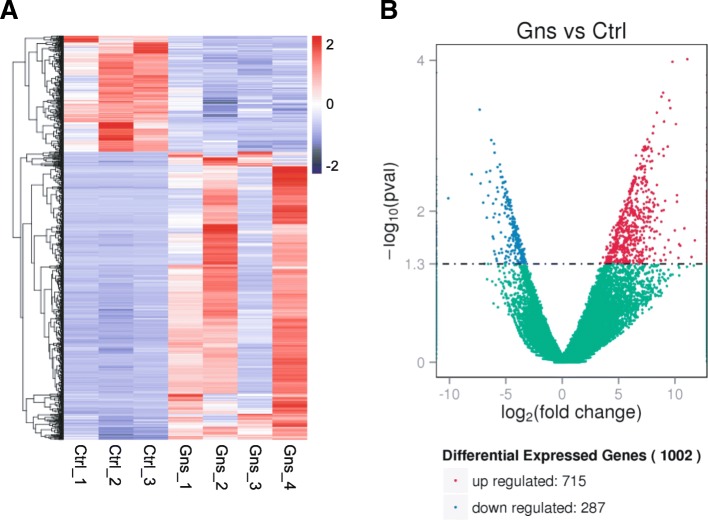
Cluster and filter analysis of DEGs. **a** Heatmap of the differentially expressed genes (DEGs) between Gns and Ctrl group. The color key from blue to red indicates the relative gene expression level from low to high, respectively. **b** Volcano plot showing DEGs. The x-axis shows the fold-change in gene expression, and the y-axis shows significant statistical differences. Red, up-regulated genes; green, down-regulated genes; blue, genes with no significant difference in expression

We then analyzed the DEGs between the two groups. As shown in Fig. 1a, we came up with the unsupervised hierarchical clustering of DEGs. Compared with the control group, 715 genes were up-regulated, whereas 287 genes were down-regulated in the Gn-stimulated group (*P* < 0.05; FC of log_2_-transformed FPKM > 1) (Fig. 1b). The detailed regarding genes that were up-regulated and down-regulated in each group are presented in Additional file 3: Table S2 and Additional file 4: Table S3.

**Fig. 2:**
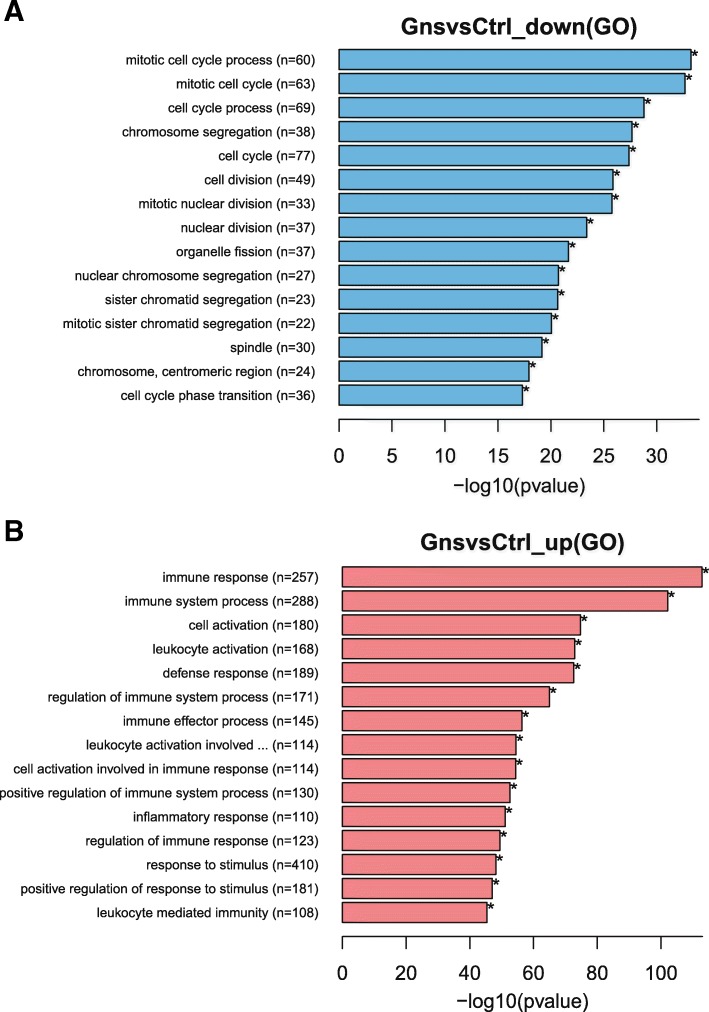
GO enrichment in biological process analysis of DEGs. The x-axis shows −log_10_ (*P*-value), and the y-axis shows five representative GO biological process terms and the number of genes representing each category (n). **a** Genes down-regulated in Gns compared with the Control; **b** Genes up-regulated in Gns compared with the Control

To investigate the possible biological functions of DEGs between the two groups, we performed GO analysis of DEGs and identified enriched biological processes in each group (the detailed information is presented in Additional file 5: Table S4 and Additional file 6: Table S5). The top 15 GO enrichment results are shown in Fig. 2a (the down-regulated DEGs) and Fig. 2b (the up-regulated DEGs). The DEGs down-regulated in the Gn-stimulated group were significantly enriched and related to the cell cycle and chromosome segregation. In contrast, the up-regulated DEGs were enriched in functions that are related to immune response or processes.

**Fig. 3:**
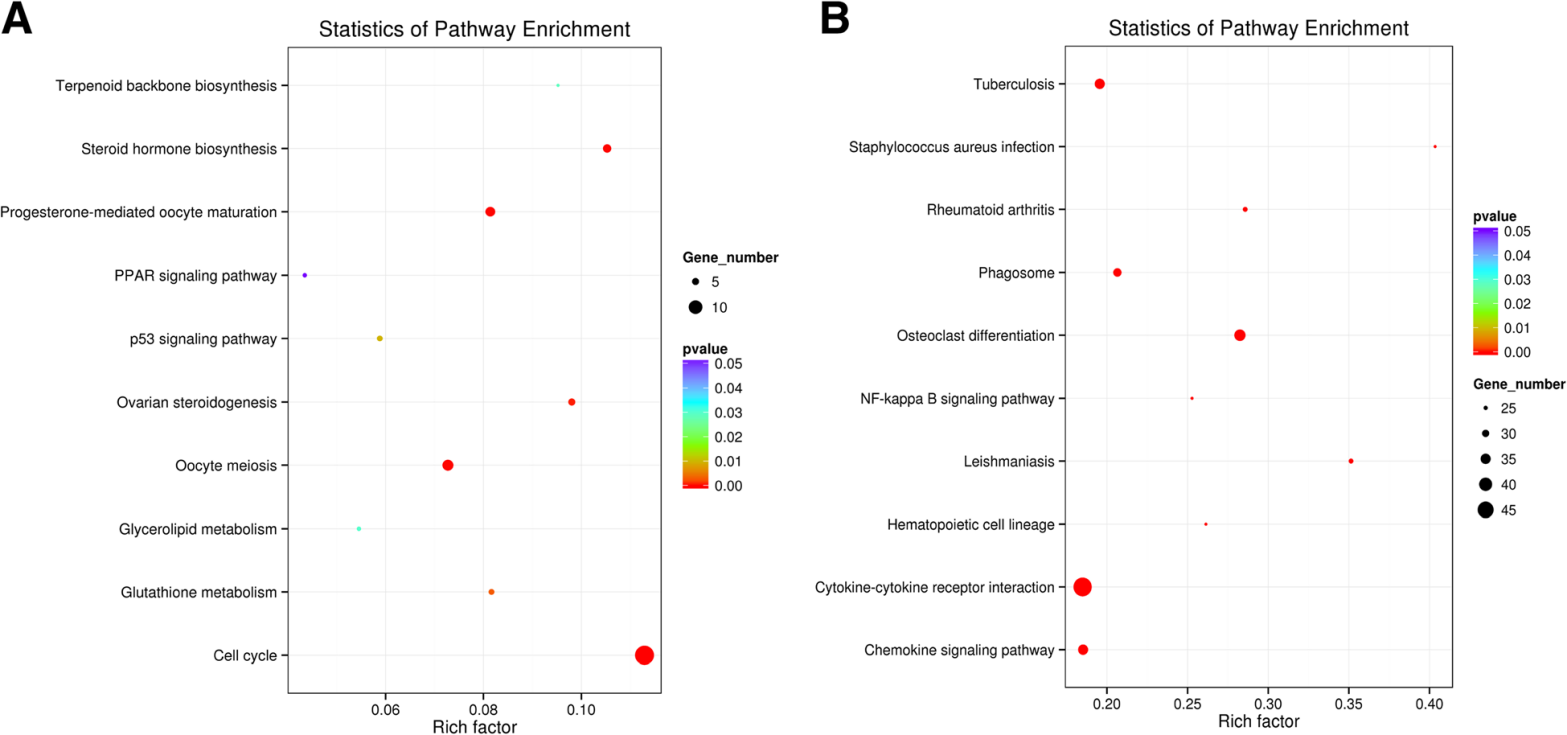
KEGG enrichment analyses of DEGs. The ratio of the number of DEGs to the total gene number is represented by the enrichment factor. Size of dots: number of genes; color of dots: range of *P*-values. **a** Representative pathways of down-regulated genes in Gns group vs. Control group; **b** Representative pathways of up-regulated genes in Gns group vs. Control group

Next, we performed the KEGG analysis to investigate the differences between the two groups. Detailed pathway enrichment analyses of down-regulated and up-regulated DEG data are presented in Additional file 7: Table S6 and Additional file 8: Table S7, respectively. The representative top 10 pathways (*P* < 0.05) in DEGs that were down-regulated in the Gn-stimulated group are shown in Fig. 3a. Amongthem, only five pathways (cell cycle, oocyte meiosis, steroid hormone biosynthesis, P-mediated oocyte maturation, and ovarian steroidogenesis) were significantly enriched (adjusted *P* < 0.05). The names of the DEGs in each pathway are annotated in Table 1. On the other hand, among the up-regulated genes in the Gn-stimulated group, 38 pathways were enriched (adjusted *P* < 0.05). The top 10 terms are shown in Fig. 3b, including three representative terms (cytokine-cytokine receptor interaction, chemokine signaling pathway, and NF-kappa B signaling pathway), with the correlated input genes annotating in Table 1.

**Table 1:** Canonical pathways enriched (*P* < 0.001) among genes down-regulated or up-regulated in the Gns group versus the Control group

Pathway	Input number	Background number	Corrected *P*-value	Genes
Pathways enriched among down-regulated genes				
Cell cycle	14	124	1.10E-07	PKMYT1, PLK1, CDC25A, TTK, CDC20, CDC25C, CCNA2, CCNB2,
Oocyte meiosis	8	110	0.003	BUB1B, CDC45, CCNB1, CDC6, ORC6, ESPL1 PKMYT1, PLK1, CDC20, CDC25C, CCNB2, AURKA, CCNB1, ESPL1
Steroid hormone biosynthesis	6	57	0.003	CYP19A1, HSD11B2, CYP17A1, CYP21A2
Progesterone-mediated oocyte maturation	7	86	0.003	PKMYT1, PLK1, CDC25A, CCNB2, CDC25C, CCNA2, CCNB1
Ovarian steroidogenesis	5	51	0.0125	FSHR, CYP19A1, CYP17A1, LHCGR
Pathways enriched among up-regulated genes				
Cytokine–cytokine receptor interaction	49	265	2.68E-10	TNFSF11, CCL28, IL1B, CCR2, CXCL3, OSM, PPBP, TNF, IL10, TNFSF8, CCL4, CXCL8, CXCR4, CD40, IL10RA, IL1R1, FLT3, CCR1, LTA, CCR5, TNFRSF11A, TGFBR2, CCL4L2, TNFRSF18, CCL22, CXCL2, CCL8, TNFSF10, CCL2, CCL20, IL6R, CSF2RA, CCL3L1, TNFSF14, TNFRSF8, TNFSF13B, IL1R2, IL6, CSF2RB, CCL3, CCR6, TNFRSF4, CRLF2, CCL7, IL18R1, IL18RAP, TNFRSF1B, IL18, CSF3R
Chemokine signaling pathway	35	189	9.0E-08	CCL28, WAS, CCR2, CXCL3, PRKCB, PPBP, CCL4, HCK, CXCL8, CXCR4, PIK3R5, DOCK2, CCR1, CCR5, ARRB2, CCL4L2, CCL22, CXCL2, CCL8, CCL2, CCL20, CCL3L1, ADCY7, CCR6, VAV1, ADCY4, FGR, CCL3, PIK3CG
NF-kappaB signaling pathway	23	91	1.4E-07	CCL7, NCF1, PLCB2, GNGT2,TNFSF13B, PLAU, PRKCB, TNF, BLNK, BTK, CCL4, CXCL8, CD40, LTA, BCL2A1, TNFRSF11A, CCL4L2, CXCL2, ICAM1, CD14, SYK, TNFSF14, IL1B, IL1R1, TNFSF11, LY96, GADD45B

**Fig. 4:**
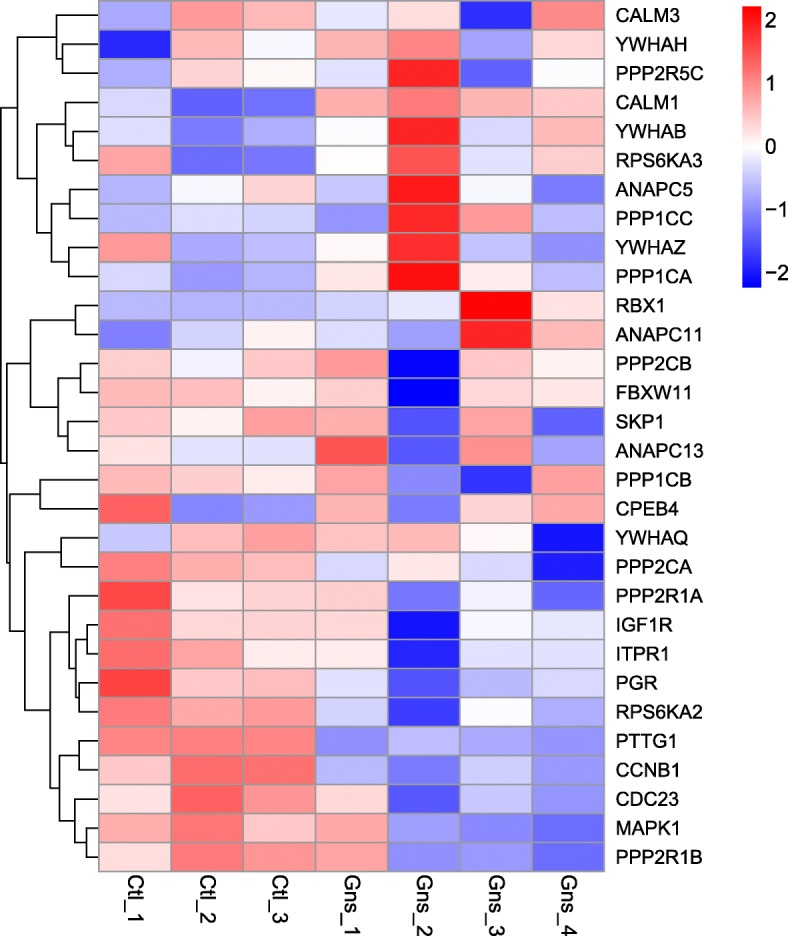
Heatmap of selected genes involved in oocyte meiosis that were differentially expressed in the Control and Gns groups. Color from blue to red indicates the relative gene expression level from low to high

To further investigate whether oocyte meiosis was affected by Gn stimulation, we clustered some mitosis–associated genes and examined their expression among the seven samples (Fig. 4). This analysis revealed that some cell cycle– or mitosis-associated genes were expressed at low levels in the Gn-stimulated group, including *CCND2*, *CCNB1*, *CDC23*, *CDK1*,and *SMAD3*. Moreover, BUB3 (a mitotic checkpoint gene) was also expressed at low levels in the Gn-stimulated group. Intriguingly, HDAC2 (a member of the histone deacetylase family) was also down-regulated in the Gn-stimulated group, which forms transcriptional repressor complexes by associating with many different proteins, and thus plays an important role in transcriptional regulation, cell cycle progression, and developmental events.

### Hormonal concentrations in follicular fluid

Next, we verified different hormone concentrations in the FF samples of every single pre-ovulatory follicle used for the transcriptome analysis. The sizes of the pre-ovulatory follicles were similar between the two groups. Table 2 shows the different follicular hormonal concentrations. The LH and E2 concentrations in FF were lower in the Gn-stimulated group than in the control group (*P* = 0.006 and *P* = 0.002, respectively). However, the P4 concentration was higher in the Gn-stimulated group (*P* = 0.003). The levels of the other four hormones tested were not significantly different between the two groups.

**Table 2:** Comparison of hormonal concentration in follicular fluid aspirated respectively from the Gns and Control groups

Parameters	Control	Gns	*P*-value
Follicular diameter (cm)	1.8 ± 0.2	2.0 ± 0.1	ns
FSH (mIU/ml)	1.7 ± 1.0	7.4 ± 7.3	ns
LH (mIU/ml)	4.7 ± 1.7	1.0 ± 0.3	< 0.01
E2 (nmol/l)	5970.4 ± 910.5	1119.5 ± 1088.4	< 0.01
P4 (μmol/l)	7.3 ± 6.5	25.4 ± 2.4	< 0.01
T (nmol/l)	43.7 ± 27.2	12.3 ± 5.3	ns
AND (nmol/l)	383.7 ± 189.8	163.4 ± 120.8	ns

mIU/ml, milli-international units per milliliter

All the values are means ±SD (standard deviation). *P*-values were calculated using Student’s t test. *ns* not significant

## Discussion

In this paper, we compared the transcriptomes of granulosa cells from one leading pre-ovulatory follicle obtained from the control and Gn-stimulated groups. The results showed that DEGs down-regulated in the Gn-stimulated group (COS-stimulated) were enriched in functions related to the cell cycle or mitosis, whereas up-regulated genes were mainly involved in immune functions and cytokine–cytokine receptor interactions.

Using a microarray technique, a previous study analyzed the transcriptomes of the cumulus cells obtained from human pre-ovulatory follicles in stimulated and unstimulated cycles and found that only 18 genes were significantly differentially expressed [13]. In that study, recombinant chorionic Gn (rCG) was administered to trigger ovulation during an unstimulated cycle. However, the control group assigned in our study did not apply any stimulation intervention, which represents a completely natural status. Moreover, we detected far more DEGs (715 up-regulated and 287 down-regulated genes) in the luteinized granulosa cells.

The expression levels of *FSHR* (FSH receptor), *LHCGR* (LH receptor), and *INHBA* (inhibin A) were decreased, which reflected a unique feature of the granulosa cells before and after ovulation triggered in the COS cycle [14]. The decreases in the expression levels of *HSD11B2* (hydroxysteroid 11b dehydrogenase 2, which promotes cortisone production from cortisol), *CYP171*(which catalyzes androgen production), and *CYP19A*(which converts AND to estrogen) were accompanied by the changes in hormonal levels in FF, indicating a condition shifting from the estrogenic follicles to progesterogenic follicles under Gns stimulation.

The down-regulated genes were enriched in functions related to cell cycle andmiosis or meiosis, both of which are involved in the maturation processes of follicles and oocytes. This finding suggests that Gns can stimulate the maturation of granulosa cells by affecting the cell cycle from the precocious maturation. These results implied adecrease in GC proliferation after Gns stimulation. The significant down-regulation of genes associated with chromosome segregation and the mitotic spindle is consistent with the concept that Gns increase aneuploidy in oocytes [15] and spindle abnormalities [13]. For example, PRC1 (a protein regulator of cytokinesis 1) was down-regulated, which potentially impairs the completion of the first meiotic division [16]. Taken together, these observations confirmed the concept that granulosa cells grow more quickly when approaching ovulation but may have been insufficiently mature after Gns stimulation.

On the other hand, the up-regulation of immune and inflammation-associated genes in the Gn-stimulated group was consistent with the theory that ovulation is an acute inflammatory reaction occurs in the ovarian tissue [17]. Consistent with the results obtained from previous studies, several ovulation-associated factors such as *PTGS1* (prostaglandin endoperoxide synthetase 1), *RGS1, RGS16* (a regulator of G-protein signaling 1 and 16),*PDE2A* (3′5’-cyclic nucleotide phosphodiesterase 2A), and *ADAMTS1* (a disintegrin-like and metalloprotease with thrombospondin type 1 motif, 1) were all up-regulated in our study [14, 18].

Immune cell-derived cytokines and chemokines play important roles in regulating ovarian function [19]. Gns induce the local distribution of immune cells to release intrafollicular cytokines, which may then, in turn, affect the oocyte quality [20]. Levels of cytokines such as IL1β, IL6, and TNF-α were elevated in FFfollowing controlled ovarian hyperstimulation (COH) [21]. In the present study, we also observed significantly up-regulated expression levels of IL10, IL6, IL18, and TNF. Cytokine-cytokine receptor interactions and the TNF signaling pathway were all functionally enriched among up-regulated DEGs. Inconsistent with the results obtained from a previous study, Baskindet al. [22], reported that the majority of circulatory cytokines, such as IL8, were present at higher concentrations in the modified natural-cycle cohort than in the COH group. Kollmann et al. [20] also reported that FF contained a marginally lower concentration of IL8 under a stimulated cycle than a natural cycle. The reason for this discrepancy in these two studies may be that HCG was administered to their natural-cycle cohorts.

Two previous studies have compared the FF hormone profiles of the natural IVF cycle (with HCG administration) and the conventional stimulated IVF cycle and found that E2 and LH concentrations were significantly reduced in the Gn-stimulated group, which is consistent with our results [13, 23]. However, HCG was administered in the natural-cycle group as in both studies performed by de Los et al.and von Wolff M et al. Similar to the finding that there is a change before and after ovulation triggered using HCG, the concentrations of E2 and P4 were different between the two groups [14]. However, two other studies reported that E2 and P4 levels in FF did not differ significantly between stimulated and natural groups, in whichno HCG was administered to the natural-cycle group [21, 24]. In particular, we have to point out that we analyzed only the largest follicle of a population of multiple follicles, which was the clearest and free from contamination (especially blood cells). Therefore, we assure that the results of the hormonal measurement and granulosa cells transcriptome are sound.

There are some limitations throughout the current study. First, the transcriptome data were obtained from the mural granulosa cells obtained from FF, but not the cells that surround the oocyte, even though some DEGs were correlated with oocyte meiosis. Second, we aimed to observe the influence of Gns, however we were not able to exclude the effect of HCG administration, which was applied only in the Gn-stimulated group. On the other hand, data obtained from the natural cycle without hCG triggering may reflect a more physiological status and hence more meaningful. Third, we have to admit that the present sample size was limited and small, even though some of the results are conclusive.

## Conclusions

In summary, using a global transcriptome sequencing technique, we compared a set of genes that were differentially regulated in granulosa cells of the maturation processes in natural and Gn-stimulated cycles. The results obtained from the DEGs analyses suggested that the Gn stimulation may induce the decreased proliferation of granulosa cells, ovulation, and an increased differentiation process. Additionally, these changes were accompanied by the alteration of several hormonal levels in FF. These findings may improve our understanding of the maturation process of the oocyte. Most importantly, our findings indicate that the simulation with Gns may potentially induce meiotic errors of human oocytes.

## Additional files

Additional file 1: **Figure S1.** Correlation and gene expression analysis. A, Pearson correlation between samples; B, Venn diagram showing overlaps between the two groups. Purple, genes expressed distinctly in the Gns group; yellow, genes expressed distinctly in the Control group; orange, genes expressed in both groups. (PDF 627 kb)

Additional file 2: **Table S1.** Summary of sequence assembly after Illumina sequencing. (DOCX 15 kb)

Additional file 3: **Table S2.** Details of genes down-regulated in the Gns group compared with the Control group. (XLSX 36 kb)

Additional file 4: **Table S3.** Details of genes up-regulated in the Gns group compared with the Control group. (XLSX 86 kb)

Additional file 5: **Table S4.** GO enrichment of genes down-regulated in the Gns group compared with the Control group. (XLSX 420 kb)

Additional file 6: **Table S5.** GO enrichment of genes up-regulated in the Gns group compared with the Control group. (XLSX 791 kb)

Additional file 7: **Table S6.** Pathway enrichment of genes down-regulated in the Gns group compared with the Control group. (XLSX 26 kb)

Additional file 8: **Table S7.** Pathway enrichment of genes up-regulated in the Gns group compared with the Control group. (XLSX 45 kb)

## Data Availability

All data generated or analysed during this study are included in this published article [and its supplementary information files].
