# Supplementary figures and images for: Effect of exogenous gonadotropin on the transcriptome of human granulosa cells and follicular fluid hormone profiles
Source: Reprod Biol Endocrinol. 2019 Jun 24;17:49. doi: 10.1186/s12958-019-0489-4 (PMC6591892; doi:10.1186/s12958-019-0489-4)

A

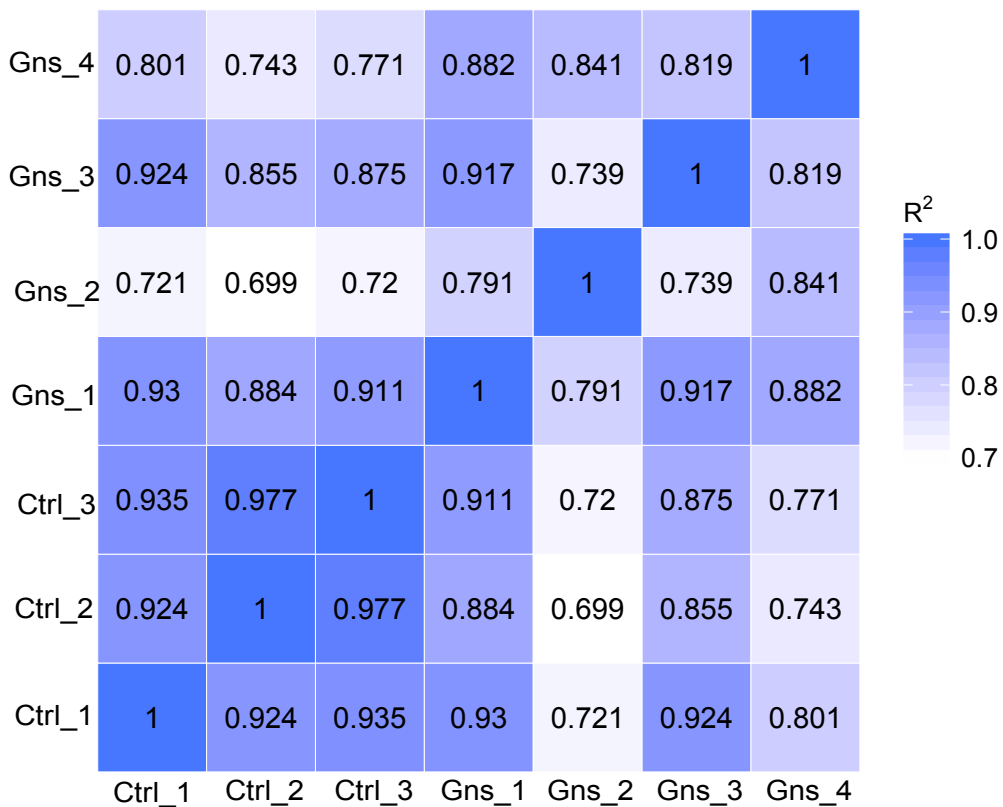

B

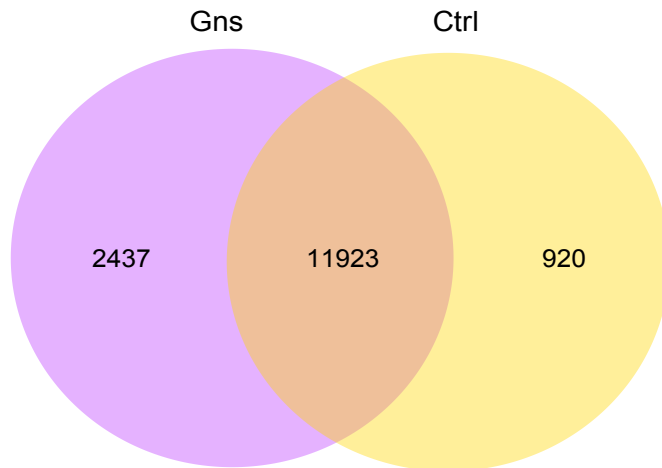

Supplement: Supplementary file 1 — Figure S1. Correlation and gene expression analysis. A, Pearson correlation between samples; B, Venn diagram showing overlaps between the two groups. Purple, genes expressed distinctly in the Gns group; yellow, genes expressed distinctly in the Control group; orange, genes expressed in both groups. (PDF 627 kb) [file 12958_2019_489_MOESM1_ESM.pdf]
