# Supplementary material for: Effect of exogenous gonadotropin on the transcriptome of human granulosa cells and follicular fluid hormone profiles
Source: Reprod Biol Endocrinol. 2019 Jun 24;17:49. doi: 10.1186/s12958-019-0489-4 (PMC6591892; doi:10.1186/s12958-019-0489-4)
Supplement: Supplementary file 2 — Table S1. Summary of sequence assembly after Illumina sequencing. (DOCX 15 kb) [file 12958_2019_489_MOESM2_ESM.docx]

**Table S1. Summary of sequence assembly after Illumina sequencing**

| **Sample** | **Raw reads** | **Clean reads** | **Clean bases** | **Error rate (%)** | **Q20 (%)** | **Q30 (%)** | **GC content (%)** |
| --- | --- | --- | --- | --- | --- | --- | --- |
| Ctrl_1 | 84937468 | 75818530 | 11.35G | 0.02 | 97.02 | 92.11 | 48.84 |
| Ctrl_2 | 85990074 | 77073658 | 11.53G | 0.02 | 97.27 | 92.67 | 48.94 |
| Ctrl_3 | 90151172 | 81093092 | 12.13G | 0.02 | 97.29 | 92.69 | 49.44 |
| Gns_1 | 82498852 | 74685944 | 11.17G | 0.02 | 97.40 | 92.92 | 50.10 |
| Gns_2 | 75648624 | 66101500 | 9.88G | 0.02 | 97.21 | 92.56 | 49.98 |
| Gns_3 | 88570480 | 78051122 | 11.67G | 0.02 | 97.50 | 93.20 | 49.45 |
| Gns_4 | 69131420 | 59153364 | 8.82G | 0.01 | 98.46 | 95.59 | 49.70 |

Notes: Q20, percentage of bases with a Phred value of at least 20; Q30, percentage of bases with a Phred value of at least 30.
